# Laboratory diagnosis of Lyme neuroborreliosis: a comparison of three CSF anti-*Borrelia* antibody assays

**DOI:** 10.1007/s10096-013-2014-6

**Published:** 2013-11-22

**Authors:** A. J. Henningsson, M. Christiansson, I. Tjernberg, S. Löfgren, A. Matussek

**Affiliations:** 1 Department of Clinical Microbiology, Ryhov County Hospital, 551 85 Jönköping, Sweden; 2 Department of Clinical Chemistry, Kalmar County Hospital, Kalmar, Sweden

## Abstract

The diagnosis of Lyme neuroborreliosis (LNB) requires the detection of intrathecal synthesis of *Borrelia*-specific antibodies, but in very early disease, the sensitivity may be low. We compared the performance of the second-generation IDEIA Lyme Neuroborreliosis test (Oxoid), based on purified native flagellum antigen, with two newly developed tests based on several recombinant antigens for the diagnosis of LNB. Patients investigated for LNB during 2003 through 2007 were included (*n* = 175); 52 with definite LNB, four with possible LNB and 119 non-LNB patients. Serum and cerebrospinal fluid (CSF) were analysed with the IDEIA Lyme Neuroborreliosis (Oxoid), VIDAS Lyme IgG (bioMérieux) and recomBead Borrelia IgM and IgG (Mikrogen) assays. Intrathecal antibody indices (AIs) were calculated according to the manufacturers’ protocols. The IDEIA test performed with an overall sensitivity (IgM and IgG AIs taken together) of 88 % and a specificity of 99 %. The VIDAS test showed a sensitivity of 86 % and a specificity of 97 %. An overall sensitivity of 100 % and a specificity of 97 % were achieved by the recomBead test. We conclude that the three assays performed equally well regarding specificity, but our data suggest an improved diagnostic sensitivity with the recomBead Borrelia test.

## Introduction

The tick-transmitted disease Lyme borreliosis is caused by spirochetes belonging to the *Borrelia burgdorferi* sensu lato (s.l.) genospecies complex. Three of the genospecies are most frequently isolated from human specimens: *B*. *afzelii*, *B*. *garinii* and *B*. *burgdorferi* sensu stricto (s.s.). Occasionally, other genospecies have been associated with human disease, e.g. *B*. *spielmanii* [1].

Lyme neuroborreliosis (LNB) is the most common manifestation of disseminated borreliosis in Europe [2–4]. The symptoms and the disease course of LNB differ between individuals, which is partly assumed to depend on which genospecies causes the infection, e.g. *B*. *garinii* has been associated with more distinct symptoms and more pronounced intrathecal inflammation than *B*. *afzelii* [5].

The diagnosis of LNB is, according to current European guidelines [6], based on the patient’s medical history, clinical findings and analysis of cerebrospinal fluid (CSF) with confirmation by culture, polymerase chain reaction (PCR) or specific anti-*Borrelia* antibody index (AI). The sensitivity of culture and PCR in clinical specimens is, however, low (10–30 %) [7–9], and, consequently, these methods are of limited use. The detection of an elevated anti-*Borrelia* AI remains the main confirmatory tool in LNB diagnostics. Rapid and easy-to-use tests delivering clear-cut results are important for laboratories analysing large numbers of samples. A rapid and reliable diagnosis of LNB is essential for patients, since delayed antibiotic treatment is associated with slower recovery and persistent symptoms [10, 11].

First-generation anti-*Borrelia* antibody tests were based on whole-cell sonicates and had poor specificity due to cross-reactive antibodies [9, 12, 13]. The second generation of antibody tests, based on purified native *Borrelia* antigens such as the flagellum protein, have improved the specificity [13]. Now, third-generation antibody tests based on synthetic peptides and recombinant antigens are available [12], and the use of these tests might further improve both the sensitivity and specificity in LNB diagnostics.

The aim of this study was to compare the diagnostic performance of the second-generation IDEIA Lyme Neuroborreliosis test (Oxoid, Hampshire, UK), currently in use in our laboratory, but with a limited sensitivity in very early LNB [14], with two third-generation antibody assays based on several recombinant antigens for the laboratory diagnosis of LNB. Since comparisons of antibody assays are often complicated by the lack of gold standards, much effort was directed to the definition and characterisation of the included patients.

## Materials and methods

### Study populations and clinical specimens

Serum and CSF specimens were selected retrospectively from 175 clinically well-characterised individuals who had been investigated for suspected LNB from 2003 through 2007 in Jönköping County, Sweden (Table 1). Fifty-two patients had definite LNB according to the European guidelines [6]; neurological symptoms consistent with LNB (one or several of the following symptoms: headache/neck pain *n* = 35; cranial nerve palsy *n* = 33; muscle/joint pain *n* = 27; radiculitis *n* = 22; paresthesia *n* = 22; vertigo *n* = 6), CSF pleocytosis (mononuclear cell count >5/μL) and elevated anti-*Borrelia* AI. The Lyme Borreliosis ELISA kit 2nd Generation (Dako Cytomation A/S, Glostrup, Denmark), which is based on purified native flagellum from *B*. *burgdorferi*, was used as the routine method for both serum and CSF at the Department of Clinical Microbiology, Ryhov County Hospital, Jönköping, Sweden, during the sampling period. The *Borrelia*-specific AI was calculated as described by Peter [15], with the modification that total IgG was substituted for *Rubella*-specific IgG. The formula used was: [*Borrelia*-specific IgG in CSF (OD)/*Borrelia*-specific IgG in serum (OD)]/[*Rubella*-specific IgG in CSF (OD)/*Rubella*-specific IgG in serum (OD)]. From September 2004, the laboratory used total IgG as the reference molecule. Eight of the patients in the definite LNB group had *Borrelia*-specific IgM alone in serum with the Lyme Borreliosis ELISA kit 2nd Generation, 11 patients had only IgG in serum, 26 had IgM and IgG, and seven had no *Borrelia*-specific antibodies detectable in serum. The LNB patients were clinically evaluated regarding symptoms, course of the disease and response to therapy by review of their medical records according to a standardised protocol. Furthermore, they had been part of previous studies [11, 16, 17], and were characterised regarding cytokine and chemokine patterns in serum and in CSF. All of the LNB patients, except four (48/52), displayed high CSF levels (>500 pg/mL) of the B cell attractant chemokine CXCL13, which has recently been shown to be a reliable marker of active LNB [18, 19]. The four LNB patients with lower CXCL13 levels in CSF (<7.8–363 pg/mL) presented with neurological symptoms (at least two of the following: headache/neck pain *n* = 2; muscle/joint pain *n* = 3; radiculitis *n* = 2; paresthesia *n* = 1; vertigo *n* = 1; cognitive dysfunction *n* = 1) and responded well to antibiotic treatment. Twenty-two of the 52 patients with definite LNB were children under 18 years of age, comprising 15 boys and seven girls.

**Table 1:** Characteristics of the patient groups

		CSF^a^ mononuclear cells >5/μL	CSF anti-*Borrelia* antibody index	Serum anti-*Borrelia* antibodies	Duration of symptoms (days): median; range	Males (%)	Age (years): median; range
LNB patients	Definite LNB^b^, *n* = 52	+	+	+/−	21; 1–224	64	39; 3–85
	Possible LNB, *n* = 4	+	−	+	9; 2–21	75	30; 4–49
Non-LNB patients	Pleocytosis for other reason, *n* = 29	+	−	+/−	7; 1–1,092	41	38; 17–80
	No pleocytosis, *n* = 90	−	−	−	n.d.^c^	38	45; 11–89

^a^ *CSF* cerebrospinal fluid

^b^ *LNB* Lyme neuroborreliosis

^c^ *n.d.* not determined

In addition, four patients with possible LNB were included; one adult with radiculitis and more than a two-fold increase of anti-*Borrelia* antibody levels in serum at follow-up, one child with facial palsy who had an erythema migrans 3 weeks earlier, one adult with meningitis and facial palsy, and one child with facial palsy. They all had CSF pleocytosis and a recent onset of their symptoms at presentation (2 days–3 weeks), but an elevated anti-*Borrelia* AI was not detected by the Lyme Borreliosis ELISA kit 2nd Generation. Three of the patients had both *Borrelia*-specific IgM and IgG in serum, and one patient had only IgG. Three of the patients had high levels of CXCL13 in their CSF (>500 pg/mL). All of them received antibiotic treatment (ceftriaxone or doxycycline) and responded promptly to therapy.

Serum and CSF samples from 119 non-LNB patients were included as a reference group. Twenty-nine of these patients had CSF pleocytosis, but no elevated anti-*Borrelia* AI was detected by the Lyme Borreliosis ELISA kit 2nd Generation. Six of these patients had positive or equivocal results for IgM in serum with this test, one patient had *Borrelia*-specific IgG and the remaining 22 patients had no detectable anti-*Borrelia* antibodies in serum. Within this group, ten patients were diagnosed with viral meningitis, seven with multiple sclerosis, three with cerebral tumour, one with *Mycoplasma pneumoniae* encephalitis, one with endocarditis lenta, one with drug intoxication, one with Bell’s palsy, one with migraine and sciatica, three with suspected autoimmune diseases and one with subarachnoid haemorrhage. The remaining 90 patients had a normal CSF cell count, a normal CSF:serum albumin ratio and no detectable anti-*Borrelia* antibodies in CSF or serum using the Lyme Borreliosis ELISA kit 2nd Generation.

Furthermore, serum samples from 90 healthy blood donors (male:female ratio = 47:42, age = 30–61 years, median 46 years) were analysed with the VIDAS Lyme IgG and IgM assay and the recomBead Borrelia IgG and IgM assay, and used for comparison with the serum results in our non-LNB group.

Serum and CSF samples had been stored at −20 °C.

### Methods

Serum and CSF specimens were tested in parallel with three antibody assays. IDEIA Lyme Neuroborreliosis is an enzyme immunoassay (EIA) based on purified native flagellum from *B*. *afzelii* strain DK1. This test determines intrathecally produced anti-*Borrelia* IgM and IgG, with no need for correction of passive transudation of serum antibodies. VIDAS Lyme IgG (bioMérieux, Marcy l’Etoile, France) is a random access enzyme-linked fluorescent assay (ELFA) based on the recombinant *Borrelia* proteins variable major protein-like sequence-expressed (VlsE), decorin-binding protein A (DbpA) and outer surface protein C (OspC). Only anti-*Borrelia* IgG is measured in CSF specimens with this test, and the AI is calculated for all antigens together in relation to either the CSF:serum albumin ratio or the CSF:serum total IgG ratio [20, 21]. The recomBead Borrelia IgM and IgG assay (Mikrogen GmbH, Neuried, Germany) is a multiplex bead array using Luminex xMAP technology and, in this study, a Bio-Plex 200 System (Bio-Rad Laboratories, Inc., Hercules, CA, USA) was used together with the software Xponent, version 3.1.871.0 (Luminex Corporation, Austin, TX, USA). The test includes several recombinant *Borrelia* antigens attached to polystyrene beads: p100 from *B*. *afzelii*, VlsE fusion protein representing different genospecies, p58 from *B*. *garinii*, p39 from *B*. *afzelii*, OspA from *B*. *afzelii*, OspC from *B*. *burgdorferi* s.s., *B*.*afzelii* and *B*. *garinii*, p18/DbpA from *B*. *burgdorferi* s.s., *B*. *afzelii*, *B*. *garinii*, *B*. *bavariensis* and *B*. *spielmanii*. Intrathecal AI is calculated for each antigen separately according to Reiber and Peter [22] by the Excel program available from Mikrogen, and an overall assessment of the test result is given. Cut-off levels and interpretation criteria were applied as recommended by the manufacturer for each test, and equivocal results were regarded as positive.

Albumin, total IgM and total IgG were measured in serum and in CSF by rate nephelometry with the Immage 800 instrument (Beckman Coulter, Fullerton, CA, USA).

### Statistics

Statistical analysis was performed using SPSS version 15.0 (SPSS Inc., Chicago, IL, USA). For pairwise comparison of the antibody assays, McNemar’s test with Yate’s correction was applied, and two-tailed *p*-values <0.05 were considered to be significant.

### Ethics

The study was approved by the Regional Ethical Review Board in Linköping, Sweden (M83-05 T91-08, 2012/246-31). Permission to read the patients’ medical records was given by the medical director of each clinic.

## Results

### Intrathecal antibody indices

The test results based on intrathecal anti-*Borrelia* IgM and IgG AIs are presented in Table 2. Overall sensitivities and specificities (IgM and IgG AIs taken together) were calculated for LNB patients (*n* = 56) and non-LNB patients (*n* = 119) (Table 2). Pairwise comparison of the overall test performances for all patients (*n* = 175) did not reveal any significant differences between the three assays. When comparing the test performances in only the LNB group (*n* = 56), the recomBead assay had significantly more positive results than the IDEIA Lyme Neuroborreliosis assay (*p* = 0.016) and the VIDAS Lyme IgG assay (*p* = 0.008). There was no significant difference between the IDEIA and VIDAS assays (*p* = 1.0).

**Table 2:** Overall test results for intrathecal anti-*Borrelia* antibody indices (AIs)

IDEIA Lyme Neuroborreliosis					
	LNB^a^ patients		Non-LNB patients		Sensitivity: 88 %^b^ Specificity: 99 %
	Definite LNB, *n* = 52	Possible LNB, *n* = 4	Pleocytosis for other reason, *n* = 29	No pleocytosis, *n* = 90	
Positive IgM and/or IgG index	48	1	1	0	
Negative IgM and IgG index	4	3	28	90	
VIDAS Lyme IgG					
	LNB patients		Non-LNB patients		Sensitivity: 86 % Specificity: 97 %
	Definite LNB, *n* = 52	Possible LNB, *n* = 4	Pleocytosis for other reason, *n* = 29	No pleocytosis, *n* = 90	
Positive IgG index	45	3	2	1	
Negative IgG index	7	1	27	89	
recomBead Borrelia IgM and IgG					
	LNB patients		Non-LNB patients		Sensitivity: 100 % Specificity: 97 %
	Definite LNB, *n* = 52	Possible LNB, *n* = 4	Pleocytosis for other reason, *n* = 29	No pleocytosis, *n* = 90	
Positive IgM and/or IgG index	52	4	3	1	
Negative IgM and IgG index	0	0	26	89	

^a^ *LNB* Lyme neuroborreliosis

^b^Sensitivity and specificity calculations are based on the LNB group (*n* = 56) and the non-LNB group (*n* = 119)

In the IDEIA Lyme Neuroborreliosis test, nine (17 %) of the 52 patients with definite LNB had elevated anti-*Borrelia* IgM AI alone, 11 (21 %) had only elevated anti-*Borrelia* IgG AI and 28 (54 %) patients had elevated AI for both anti-*Borrelia* IgM and IgG. In the recomBead Borrelia test, nine (17 %) of the patients with definite LNB had elevated anti-*Borrelia* IgG AI alone, 43 (83 %) had elevated AI for both anti-*Borrelia* IgM and IgG, but none of the patients had an elevated AI for anti-*Borrelia* IgM alone. In the possible LNB group, one of the four patients had an elevated IgM AI alone with the IDEIA test. This patient had an elevated IgG AI according to the VIDAS Lyme IgG assay, and elevated IgM and IgG AIs according to the recomBead Borrelia test. Two of the four patients with possible LNB had elevated IgM and IgG AIs with the recomBead test, and two patients had elevated IgG AIs alone.

No equivocal interval for the test results are given by the manufacturer of the IDEIA Lyme Neuroborreliosis test, whereas equivocal intervals are defined for both the VIDAS Lyme IgG and the recomBead Borrelia IgM and IgG assays. No equivocal AIs were obtained with the VIDAS Lyme IgG test, but one patient with definite LNB had an equivocal IgG AI in the recomBead test.

In the VIDAS Lyme IgG assay, the intrathecal AI was calculated based on both the CSF:serum albumin ratio and the CSF:serum total IgG ratio. When the albumin ratio was used, 43 of the 52 definite LNB cases had an elevated anti-*Borrelia* AI, and when using the total IgG ratio, an additional two patients had an elevated AI (in total, 45/52).

**Fig. 1:**
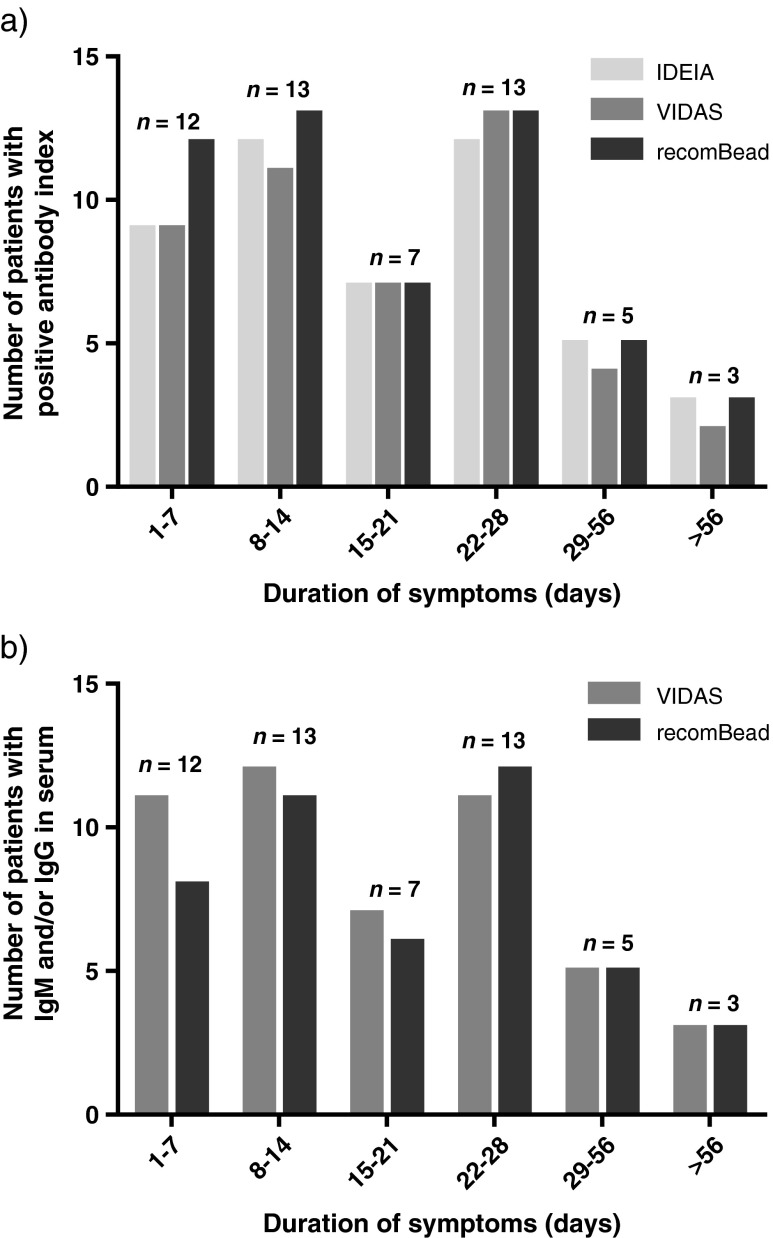
Number of definite and possible Lyme neuroborreliosis (LNB) patients with positive anti-*Borrelia* IgM and/or IgG antibody index (**a**) and anti-*Borrelia* IgM and/or IgG in serum (**b**) in each test in relation to the duration of neurological symptoms. Data on symptom duration were missing in three cases. The total number of patients (*n*) in each time interval is indicated

The number of definite and possible LNB patients with positive AI in each test in relation to the duration of neurological symptoms is presented in Fig. 1a. Data on the duration of symptoms was lacking in three of the 56 cases.

### Serum

The serum results for the VIDAS Lyme IgM and IgG and for the recomBead Borrelia IgM and IgG tests are presented in Table 3. For the VIDAS Lyme IgM and IgG assay, two patients with definite LNB had equivocal IgM results in serum and three patients in the non-LNB group had equivocal serum IgM results. As for the recomBead Borrelia IgM and IgG assay, one equivocal test result was obtained from the serum analyses; one patient with definite LNB had an equivocal value for serum IgM. The number of definite and possible LNB patients with anti-*Borrelia* IgM and/or IgG detectable in serum with each test in relation to the duration of neurological symptoms is presented in Fig. 1b.

**Table 3:** Overall test results for anti-*Borrelia* serum antibodies

VIDAS Lyme IgM and IgG					
	LNB^a^ patients		Non-LNB patients		Sensitivity: 93 %^c^ Specificity: 86 %
	Definite LNB, *n* = 52	Possible LNB, *n* = 4	Pleocytosis for other reason, *n* = 29	No pleocytosis, *n* = 90	
Positive S^b^-IgM and/or S-IgG	48	4	8	9	
Negative S-IgM and S-IgG	4	0	21	81	
recomBead Borrelia IgM and IgG					
	LNB patients		Non-LNB patients		Sensitivity: 86 % Specificity: 85 %
	Definite LNB, *n* = 52	Possible LNB, *n* = 4	Pleocytosis for other reason, *n* = 29	No pleocytosis, *n* = 90	
Positive S-IgM and/or S-IgG	45	3	7	11	
Negative S-IgM and S-IgG	7	1	22	79	

^a^ *LNB* Lyme neuroborreliosis

^b^ *S* serum

^c^Sensitivity and specificity calculations are based on the LNB group (*n* = 56) and the non-LNB group (*n* = 119)

In the recomBead Borrelia IgM and IgG test, the antibody reactivities seen in serum from the LNB patients (*n* = 56) were directed against VlsE in 100 % of the cases, OspC in 79 %, p18/DbpA in 52 %, p58 in 50 %, p39 in 46 %, p100 in 43 % and OspA in 9 %. All OspC-reactive samples showed reactivity against OspC from at least two of the included *Borrelia* species (*B*. *burgdorferi* s.s., *B*.*afzelii* and *B*. *garinii*). Sera reactive against p18/DbpA, on the other hand, generally showed reactivity against p18/DbpA from only one of the represented *Borrelia* species (*B*. *burgdorferi* s.s., *B*. *afzelii*, *B*. *garinii*, *B*. *bavariensis* and *B*. *spielmanii*), most frequently against *B*. *garinii* (79 %).

In addition, serum samples from 90 healthy blood donors were analysed with the VIDAS Lyme IgM and IgG assay and with the recomBead Borrelia IgM and IgG assay, and a seropositivity of 11 % was found with both tests. Eight of the blood donors who had positive test results with the VIDAS Lyme IgG and IgM assay also tested positive with the recomBead Borrelia IgM and IgG assay. The IgM and/or IgG seropositivity in the non-LNB group (*n* = 119) was 14 % in the VIDAS Lyme IgM and IgG test and 15 % in the recomBead Borrelia IgM and IgG test.

## Discussion

In this study, we compared the diagnostic performance of the second-generation IDEIA Lyme Neuroborreliosis test with two third-generation assays in serum and CSF from 175 clinically well-defined patients for the diagnosis of LNB. We found that, when serum and CSF were tested in parallel and AIs were calculated according to the manufacturers’ instructions, all three assays performed equally well regarding the specificity (97–99 %). However, the IDEIA Lyme Neuroborreliosis assay is based on the same antigen as the Lyme Borreliosis ELISA kit 2nd Generation that was used for the selection of the patient groups, and, thus, the specificity of the IDEIA test cannot be reliably evaluated. The IDEIA Lyme Neuroborreliosis test and the VIDAS Lyme IgG test had a sensitivity of 88 % and 86 %, respectively, whereas a sensitivity of 100 % was achieved by the recomBead Borrelia IgM and IgG test, and the difference between the recomBead test and the other two assays was statistically significant in the LNB group. When comparing the test performances for all the included patients (LNB and non-LNB), however, the differences between the tests did not reach statistical significance.

All three assays gave elevated AIs in some of the non-LNB patients, especially in the group of patients with CSF pleocytosis for other reason (Table 2). This could be due to unspecific antibody reactivities, but it is also possible that these elevated AIs are correct, since the classification of patients was based on the older Lyme Borreliosis ELISA kit 2nd Generation, and that the patients, according to their medical records, were diagnosed with conditions other than LNB. However, the negative test result of the anti-*Borrelia* antibody analysis may have influenced the clinical diagnosis. Therefore, it is possible that some of the patients classified in the non-LNB group actually had LNB. Contradicting this hypothesis, none of the six patients had positive AI in more than one of the evaluated assays.

Almost no equivocal AIs were obtained using the assays tested, which is considered advantageous for the interpretation of test results in the clinical laboratory setting. The use of the CSF:serum total IgG ratio in the calculation of AIs seems to be better than the albumin ratio when using the VIDAS Lyme IgG assay.

All of the 56 patients with definite and possible LNB were identified as having positive anti-*Borrelia* AI in the recomBead Borrelia IgM and IgG test, whereas the other two tests gave negative test results in seven and eight of the cases, respectively. Most of the patients with negative AIs in the IDEIA Lyme Neuroborreliosis test and the VIDAS Lyme IgG test had short duration of their neurological symptoms (<2 weeks), but the VIDAS assay gave negative results for two patients who presented with symptoms lasting for more than a month.

In this study, we have considered IgM AI and IgG AI as equivalent. It is our opinion that the finding of an elevated IgM AI is almost as reliable as an elevated IgG AI in LNB, especially when the medical history, clinical findings and other laboratory findings are in accordance with the diagnosis. If a patient presents with an elevated IgM AI, IgG AI or both, it probably depends on the duration of neurological symptoms, biological variations in the immune response and what antigen is used in the assay.

The analysis of anti-*Borrelia* antibodies in serum samples alone showed lower specificity in both the VIDAS Lyme IgM and IgG and the recomBead Borrelia IgM and IgG tests. Furthermore, by using the latter test, serum analysis alone also showed a lower sensitivity than the AI. These results emphasise the importance of simultaneous analysis of serum and CSF samples for the diagnosis of LNB. In addition, we found more equivocal test results in serum than in AIs, especially with the VIDAS Lyme IgM and IgG assay.

The observation that serum samples with antibody reactivities against OspC in the recomBead Borrelia IgM and IgG test consequently displayed reactivity against OspC from at least two *Borrelia* species indicates that the response to OspC antigens overlap. However, this seems not to be the case for p18/DbpA, where antibody reactivity, generally, was directed against p18/DbpA from only one species per patient. Whether this indicates the causative *Borrelia* species in individual LNB cases remains to be investigated, perhaps by confirmation with PCR and DNA sequencing. However, this may be challenging, since these methods have low sensitivity in CSF and serum specimens.

The seropositivity rate found in the 90 blood donors corroborates previous observations of *Borrelia* seroprevalence in southern Sweden [23], and was in the same range as the non-LNB patients, thus, affirming both the specificity of the test results and the use of the non-LNB group as a reference group.

All three assays, especially the VIDAS Lyme IgM and IgG test, were easy to perform. The VIDAS instrument allows random access and, thereby, facilitates rapid test results. The calculation of AIs with both the VIDAS Lyme IgG and the recomBead Borrelia IgM and IgG tests requires the analysis of albumin and total IgG (even total IgM for the latter test) in serum and in CSF, which is a disadvantage compared to the IDEIA Lyme Neuroborreliosis assay in terms of cost and ease of use. The recomBead Borrelia IgM and IgG assay is the most expensive of the three tests, and requires access to rather advanced technology (Luminex xMAP). The VIDAS Lyme IgM and IgG test also requires the use of a special instrument (VIDAS from bioMérieux, Marcy l’Etoile, France), whereas the IDEIA Lyme Neuroborreliosis test does not. The *Borrelia*-specific AI is calculated manually from the VIDAS Lyme IgG results in serum and in CSF and from total IgG or albumin in serum and in CSF. Another disadvantage with the test may be the lack of anti-*Borrelia* IgM analysis in CSF specimens, which possibly decreases the sensitivity of the test in very early LNB [24].

In conclusion, the analysis of anti-*Borrelia* antibodies simultaneously in serum and in CSF is essential for the accurate diagnosis of LNB. The three assays compared in this study performed equally well regarding their specificity, but the data suggest an improved diagnostic sensitivity with the recomBead Borrelia test.
